# The interaction between STAT3 and nAChRα1 interferes with nicotine-induced atherosclerosis via Akt/mTOR signaling cascade

**DOI:** 10.18632/aging.102296

**Published:** 2019-10-14

**Authors:** Shuang Xu, Huaner Ni, Hangwei Chen, Qiuyan Dai

**Affiliations:** 1 Department of Cardiology, Shanghai General Hospital, School of Medicine, Shanghai Jiao Tong University, Shanghai 200080, China

**Keywords:** atherosclerosis, nicotine, nAChRα1, STAT3

## Abstract

During atherosclerosis development, nicotine and its α1 nicotinic acetylcholine receptors (nAChRα1) activate atherogenic inflammation. However, the effect of signal transducer and activator of transcription 3 (STAT3)-related inflammatory pathways in nicotine-induced atherosclerosis has been poorly studied. This study investigated the transcriptional mechanism of STAT3 in nicotine/nAChRα1-induced atherosclerosis. In vivo, *ApoE^-/-^* mice were used to establish an atherosclerotic model. Plaque area and composition were assessed by oil red O staining and immunohistochemistry. In vitro, vascular smooth muscle cells and macrophages were used to investigate cell migration, proliferation, inflammation and related signaling pathways by Transwell migration assay, EdU assay, immunofluorescence, western blotting, coimmunoprecipitation and chromatin immunoprecipitation. nAChRα1 knockdown significantly decreases the nicotine-induced upregulation of p-STAT3, p-Akt and p-mTOR in vitro, while nAChRα1 overexpression has the opposite effects. The inhibition of STAT3 attenuated nicotine-induced atherosclerosis, by reducing the proliferation and migration of vascular smooth muscle cells and inflammation in macrophages. Moreover, there is a direct interaction between STAT3 and nAChRα1 that modulates STAT3 nuclear translocation and its binding to the Akt promoter region upon nicotine exposure. Taken together, STAT3 and nAChRα1 blockade attenuates nicotine-induced atherosclerosis by reducing the migration and proliferation of vascular smooth muscle cells and inflammation in macrophages via the Akt/mTOR pathway.

## INTRODUCTION

Cardiovascular disease (CVD) has been a leading threat to human health in recent decades [1]. Atherosclerosis, one of the most common cardiovascular diseases, is considered as an early stage of many diseases, such as coronary heart disease and stroke. The progression of atherosclerosis involves a complex network of cholesterol metabolism disorders, foam cell formation, chronic inflammation, vascular smooth muscle cells (VSMCs) proliferation and migration, endothelial dysfunction, etc [2–4]. Of the associated factors, the local inflammatory process driven by the accumulation of macrophages within lesions is the most important [5].

Smoking, the second leading risk factor for death worldwide [1, 6], has been reported to accelerate the development of atherosclerosis [7–9]. Nicotine is a major chemical ingredient of cigarette smoke, and its role in atherosclerosis has received increasing research attention. It is generally accepted that nicotine binds to nicotinic acetylcholine receptors (nAChRs) and causes a conformational change in the receptor structure that causes the ion channel to open, thereby modulating the Na^+^, K^+^, Ca^+^ ion flux [10]. nAChRs can be divided into muscle-type nAChRs and neuronal-type nAChRs and are ubiquitously expressed in various cell types [11]. The silencing of the α1 subunit is associated with an 80% reduction in myofibroblasts in the lesion, a reduction in the number of immune cells in the aortic wall, and a reduction in calcification and the extracellular matrix [12]. That is, the α1 subunit plays a direct role in modulating the proliferation and migration of VSMCs. A 78% knockdown of nAChRα1 results in less severe aortic plaque growth in mice [13], indicating a significant role for nAChRα1 in atherosclerotic progression. Our group previously determined that nicotine promotes atherosclerosis development via the nAChRα1-Calpain1-MMP2 signaling pathway, and nAChRα1 deficiency attenuates nicotine-induced proliferation of VSMCs [14]. However, there has been limited studies on the transcriptional mechanism of nicotine/nAChRα1-induced atherosclerosis. Thus, it would be interesting to investigate possible transcription factors and downstream signaling cascades.

STAT3 is an important transcription factor that mediates extracellular signals involved in growth, apoptosis and inflammation. STAT3 becomes transcriptionally activated by tyrosine phosphorylation. Activated STAT3 dimerizes, translocates into the nucleus and promotes the transcription of target genes [15]. The inflammatory areas of atherosclerotic plaque lesions show stronger staining of p-STAT3, indicating that the STAT3 activation enhances the progression of atherosclerosis [16]. Nicotine enhances the inflammation in atherosclerosis [9, 17, 18], but the role of the STAT3-related inflammatory pathway in nicotine-induced atherosclerosis is unknown. Therefore, we wanted to explore the transcriptional mechanism of STAT3 in nicotine/nAChRα1-induced atherosclerosis. In this study, we profiled the interaction between STAT3 and nAChRα1 and observed that STAT3 inhibition reduces nicotine-accelerated atherosclerosis. In addition, we also showed that STAT3 blockade attenuates nicotine-induced proliferation and migration of VSMCs and inflammation in macrophages via Akt/mTOR signaling cascade.

## RESULTS

### nAChRα1 regulates nicotine-induced STAT3, Akt and mTOR phosphorylation in MOVAS and RAW264.7 cells

**Figure 1:**
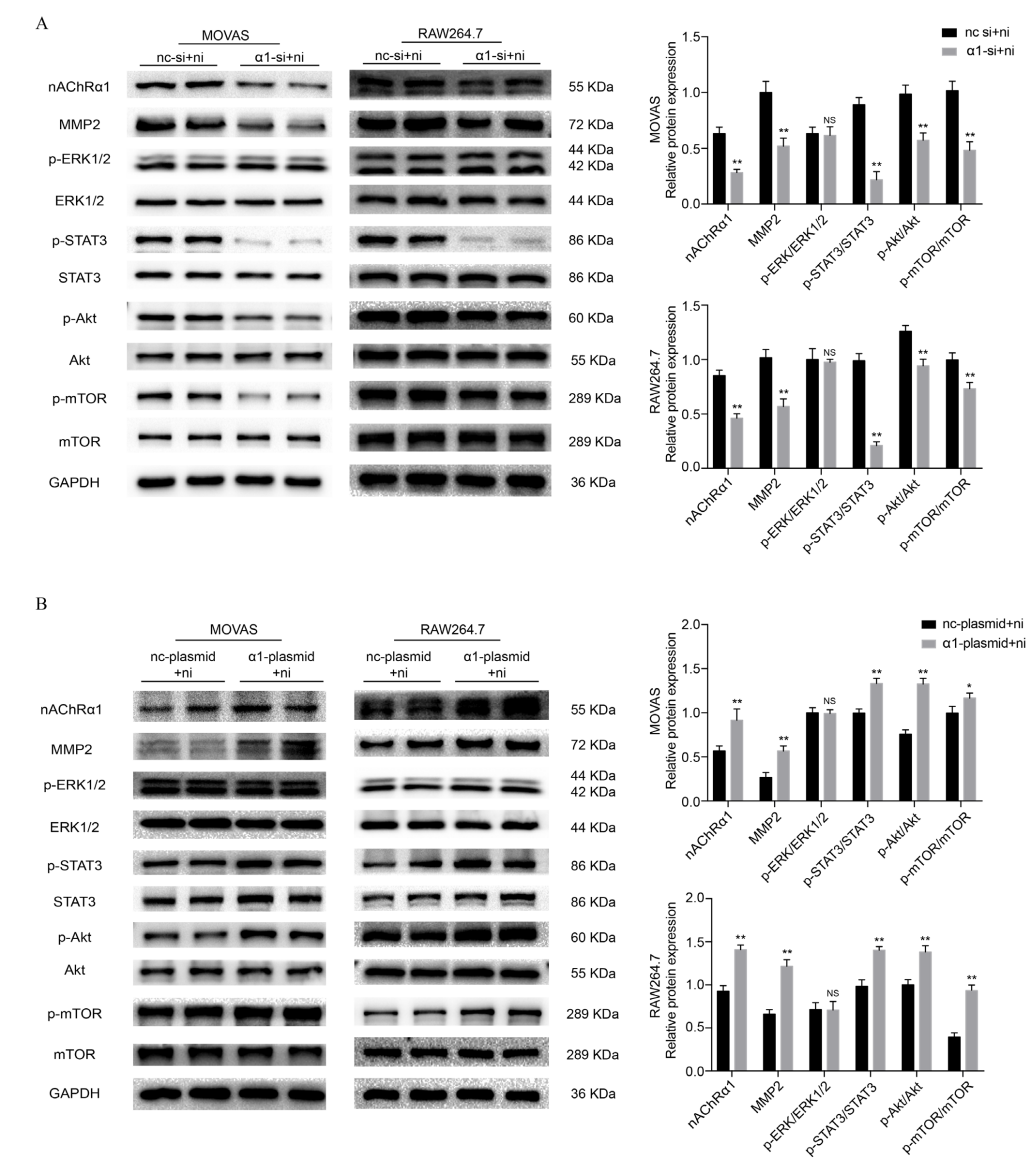
**nAChRα1 mediates the nicotine-induced phosphorylation of STAT3, Akt and mTOR in MOVAS and RAW264.7 cells.** (**A**) The effect of nAChRα1 knockdown on the protein expression and phosphorylation of ERK1/2, STAT3, Akt and mTOR in MOVAS cells (left) and RAW264.7 cells (right). (**B**) The opposite effect of nAChRα1 overexpression on ERK1/2, STAT3, Akt and mTOR in MOVAS cells (left) and RAW264.7 cells (right). Abbreviations: nc-si+ni, negative control siRNA plus nicotine; α1-si+nicotine, nAChRα1 siRNA plus nicotine; nc-plasmid+nicotine, negative control plasmid plus nicotine; α1-plasmid+nicotine, nAChRα1 overexpression plasmid plus nicotine. The data were presented as the mean ± SD. *p < 0.05, **p < 0.01 *vs.* the control group. NS, not significant *vs.* the control group. Each experiment was performed three times.

To investigate the underlying mechanisms of nicotine-induced atherosclerosis, the protein expression of STAT3, extracellular signal-regulated kinase1/2 (ERK1/2), protein kinase B (Akt) and mechanistic target of rapamycin kinase (mTOR) was evaluated. As in our previous study [14, 19], we stimulated MOVAS cells and RAW264.7 cells with nicotine at a concentration of 0, 10, 100, 500, 1000, and 5000 ng/ml for 6 h. Western blot analysis revealed that 100 ng/ml and 500 ng/ml nicotine resultes in a significant increase in the phosphorylation of ERK1/2, STAT3, Akt and mTOR in MOVAS cells (Supplementary Figure 1A) and RAW264.7 cells (Supplementary Figure 1B). Since our previous study demonstrated that nicotine promotes atherosclerosis through nAChRα1, we transfected MOVAS cells and RAW264.7 cells with siRNA to reduce nAChRα1 expression (The determination of the optimal siRNA targeting the nAChRα1 gene is shown in Supplementary Figure 1C and 1D). We found that the nicotine-induced activation of STAT3, Akt, and mTOR is significantly abolished by nAChRα1 siRNA transfection, whereas the protein level of p-ERK1/2 remaines unchanged in the two cell lines (Figure 1A). In addition, we also explored whether nAChRα1 overexpression had any effect on the nicotine-induced phosphorylation of ERK1/2, STAT3, mTOR and Akt. As shown in Figure 1B, the nicotine-induced upregulation of p-STAT3, p-Akt and p-mTOR is augmented by nAChRα1 overexpression in the two cell lines. These results indicated that nAChRα1 regulates the nicotine-induced activation of p-STAT3, p-Akt, and p-mTOR.

### STAT3 inhibition reduces the nicotine-induced proliferation and migration of MOVAS through the Akt/mTOR/MMP2 signaling pathway

**Figure 2:**
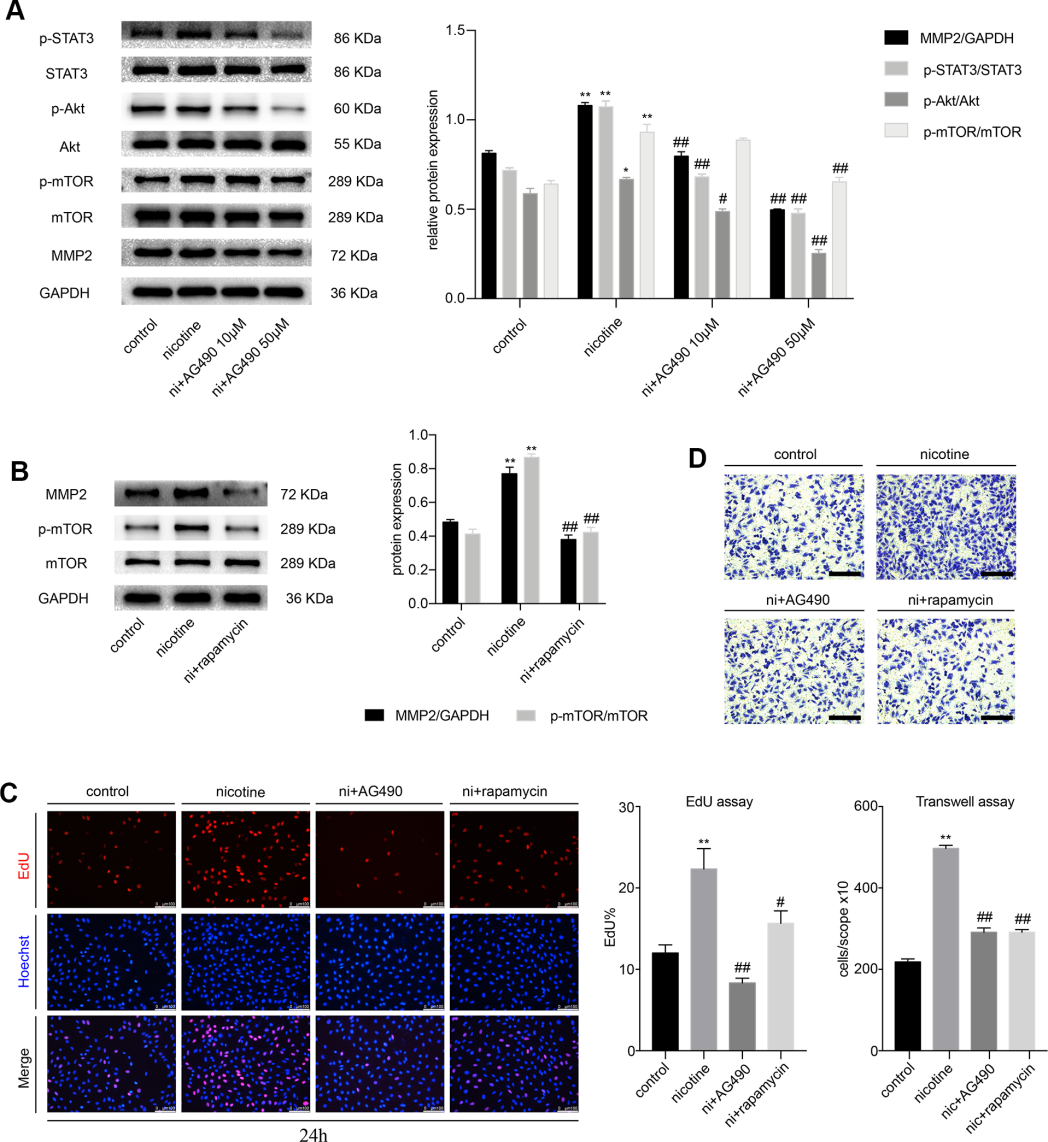
**STAT3 blockade attenuates the nicotine-induced proliferation and migration of MOVAS cells through the Akt/mTOR/MMP2 pathway.** (**A**) The effect of AG490 on the nicotine-induced activation of STAT3, Akt, mTOR and MMP2 expression in MOVAS cells. (**B**) The effect of rapamycin on the nicotine-induced upregulation of MMP2 in MOVAS cells. (**C**) The regulation of nicotine-induced proliferation by STAT3 and mTOR inhibition. Magnification, 200×; bars, 100 μm. (**D**) The regulation of nicotine-induced migration by STAT3 and mTOR inhibition. Magnification, 100×; bars, 250 μm. Ni, nicotine. The data were presented as the mean ± SD. *p < 0.05, **p < 0.01 *vs.* the control group. #*p* < 0.05, ##*p* < 0.01 *vs.* the nicotine group. Each experiment was performed three times.

Next, we pretreated MOVAS cells with AG490 (10 μM and 50 μM), a specific inhibitor of STAT3, for 6 h prior to nicotine exposure (100 ng/ml). We found that the nicotine-induced phosphorylation of STAT3, Akt and mTOR and matrix metallopeptidase 2 (MMP2) expression decreased significantly (Figure 2A). Meanwhile, we sought to determine whether Akt and mTOR were the upstream of MMP2. The specific inhibitor of mTOR, rapamycin (100 nM), was used to treat MOVAS cells for 6 h before nicotine exposure. Rapamycin downregulates nicotine-induced MMP2 expression (Figure 2B). Furthermore, we detected the proliferation and migration of MOVAS cells upon treatment with nicotine, AG490 and rapamycin by the EdU and Transwell assays. Notably, the inhibition of STAT3 and mTOR significantly reduces nicotine-induced proliferation (Figure 2C) and migration (Figure 2D). The determination of the optimal concentration of nicotine for the migration and proliferation assays is shown in Supplementary Figure 2A and 2B. These results indicated that STAT3 blockade decreases the nicotine-induced proliferation and migration of MOVAS cells through the Akt/mTOR/MMP2 signaling pathway.

### STAT3 inhibition reduces nicotine-induced inflammation in RAW264.7 cells via the Akt/mTOR/MMP2 pathway

**Figure 3:**
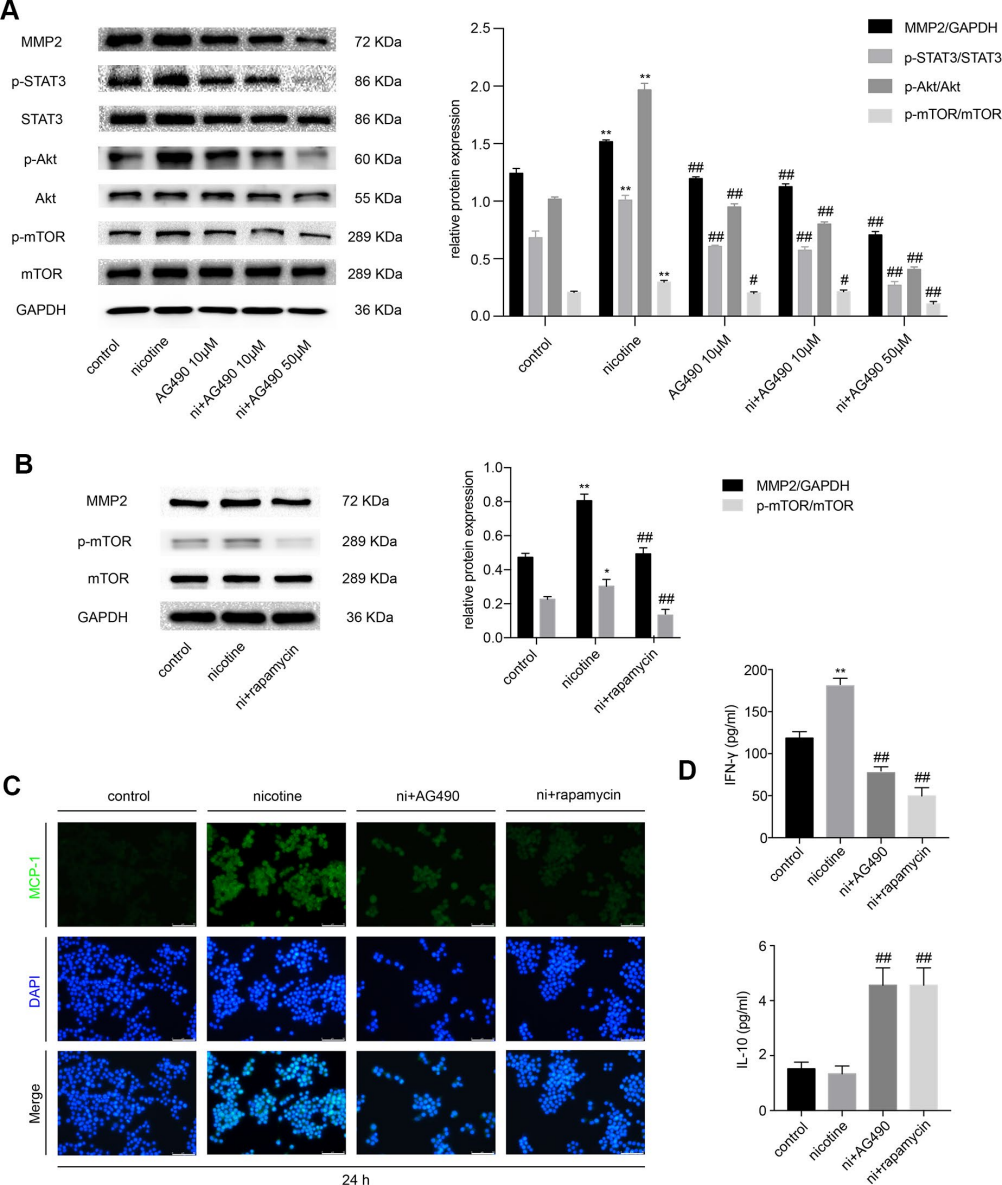
**STAT3 inhibition reduces nicotine-induced inflammation in RAW264.7 cells via the Akt/mTOR/MMP2 pathway.** (**A**) The effect of AG490 on the nicotine-induced activation of STAT3, Akt, and mTOR and the upregulation of MMP2 expression in RAW264.7 cells. (**B**) The effect of rapamycin on the nicotine-induced upregulation of MMP2. (**C**) The regulation of nicotine-induced MCP-1 expression by STAT3 and rapamycin inhibition, as detected by immunofluorescence. Magnification, 200×; bars, 100 μm. (**D**) ELISA was used to detect IFN-γ and IL-10 released by RAW264.7 cells upon treatment with nicotine, AG490 and rapamycin. Ni, nicotine. The data were presented as the mean ± SD. *p < 0.05, **p < 0.01 *vs.* the control group. #*p* < 0.05, ##*p* < 0.01 *vs.* the nicotine group. Each experiment was performed three times.

Besides, we explored whether STAT3 inactivation had any effect on nicotine-induced inflammation in RAW264.7 cells and whether Akt/mTOR/MMP2 was involved in this process. Cell treatments were the same as those in MOVAS cells. The expression of p-STAT3, p-Akt, p-mTOR and MMP2 iss remarkably decreased in the AG490 pretreatment group compared with the nicotine group (Figure 3A). Treatment with rapamycin also downregulates MMP2 expression (Figure 3B), indicating that STAT3 inhibition decreases nicotine-induced MMP2 expression through Akt/mTOR. Immunofluorescence revealed that the inhibition of either STAT3 or mTOR ameliorates the nicotine-induced upregulation of monocyte chemotactic protein 1 (MCP-1) (Figure 3C). The cell supernatant was also collected to measure the levels of inflammatory cytokines, such as interleukin-10 (IL-10) and interferon-γ (IFN-γ), by enzyme-linked immunosorbent assay (ELISA). We found that the level of the pro-inflammatory factor IFN-γ is upregulated by nicotine exposure and is reduced by AG490 and rapamycin pretreatment, and the level of the anti-inflammatory factor IL-10 is increased by AG490 and rapamycin pretreatment (Figure 3D). Taken together, AG490 remarkably attenuates nicotine-induced inflammation in RAW264.7 cells via the Akt/mTOR signaling pathway.

### A novel interaction between STAT3 and nAChRα1 occurs in MOVAS and RAW264.7 cells

**Figure 4:**
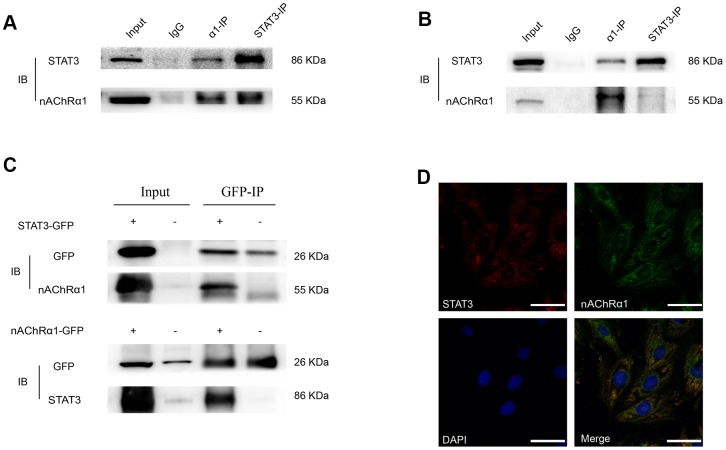
**A novel interaction between nAChRα1 and STAT3 occurs in MOVAS and RAW264.7 cells.** (**A**) Coimmunoprecipitation of nAChRα1 with STAT3 in MOVAS cells. (**B**) The direct binding of nAChRα1 and STAT3 in RAW264.7 cells. (**C**) Coimmunoprecipitation of nAChRα1 with STAT3 in 293T cells. (**D**) The colocalization of nAChRα1 and STAT3 in MOVAS cells, as determined by confocal microscopy. Magnification, 630×; bars, 50 μm. IP, immunoprecipitation, IB, immunoblot. Each experiment was performed three times.

Considering that the expression of nAChRα1 is closely related to the activation of STAT3, we hypothesized that there might be an interaction between them. To further evaluate the direct binding between STAT3 and nAChRα1, coimmunoprecipitation (CoIP) assays were performed using STAT3 and nAChRα1 antibodies. The results showed that STAT3 and nAChRα1 are substantially enriched by each other in MOVAS cells (Figure 4A) and RAW264.7 cells (Figure 4B), indicating the binding between STAT3 and nAChRα1. This binding was also confirmed in 293T cells (Figure 4C). In addition, the colocalization of nAChRα1 and STAT3 in the cytoplasm was suggested by immunofluorescence analysis under confocal microscopy (Figure 4D). This indicated that STAT3 phosphorylation might be upregulated by the interaction between STAT3 and nAChRα1 upon nicotine exposure.

### Nicotine stimulates the nuclear translocation of STAT3 and its binding to the Akt promoter region in MOVAS cells

**Figure 5:**
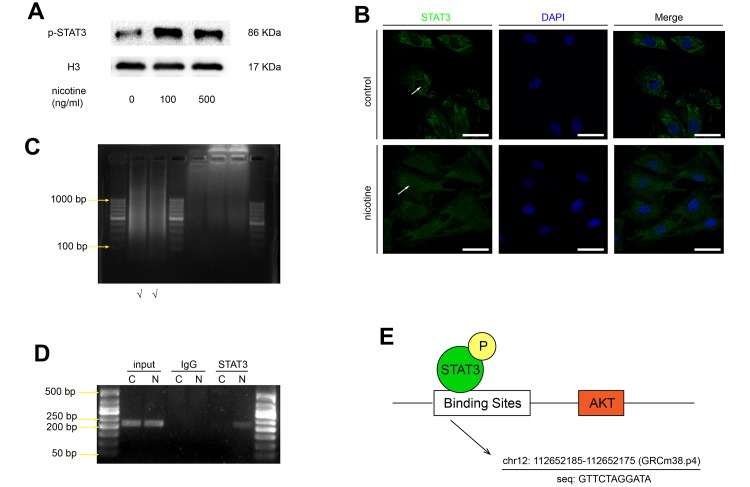
**Nicotine stimulates the nuclear translocation of STAT3 and its binding to the Akt promoter region.** (**A**) The protein expression of p-STAT3 in the nucleus in MOVAS cells upon nicotine exposure. (**B**) The nuclear translocation of STAT3 in MOVAS cells, as determined by confocal microscopy. Magnification, 630×; bars, 50 μm. Arrow means nuclear area. (**C**) The length of the DNA fragments used for the ChIP assay. (**D**) The binding of STAT3 to the promoter region of the Akt gene, as detected by the ChIP-PCR assay. (**E**) The binding site of STAT3 in the Akt promoter region, as predicted by JASPAR. Each experiment was performed three times.

It is generally acknowledged that the phosphorylated STAT3 migrates to the nucleus and binds to the promoter regions of target genes. Based on our previous finding that nicotine increases STAT3 phosphorylation, we studied whether nicotine stimulation affects the nuclear translocation of STAT3. The expression of p-STAT3 in the nucleus was detected by western blotting (Figure 5A), and the nuclear translocation of STAT3 was confirmed by confocal microscopy (Figure 5B). These results suggeste that nicotine exposure enhances the nuclear translocation of STAT3. Furthermore, chromatin immunoprecipitation-polymerase chain reaction (ChIP-PCR) assays were performed to confirm the direct binding of p-STAT3 to the Akt promoter region. The binding sites of specific transcriptional factors were predicted by JASPAR based on the GRCm38.p4 database (Figure 5E). The length of the DNA fragments used for ChIP was mainly between 200 bp and 1000 bp (Figure 5C). We found that the Akt promoter region is significantly enriched by a p-STAT3 antibody in comparison with an IgG antibody, indicating that p-STAT3 is recruited to the Akt promoter region, especially upon nicotine exposure (Figure 5D).

### STAT3 inactivation attenuates nicotine-induced plaque development in vivo

**Figure 6:**
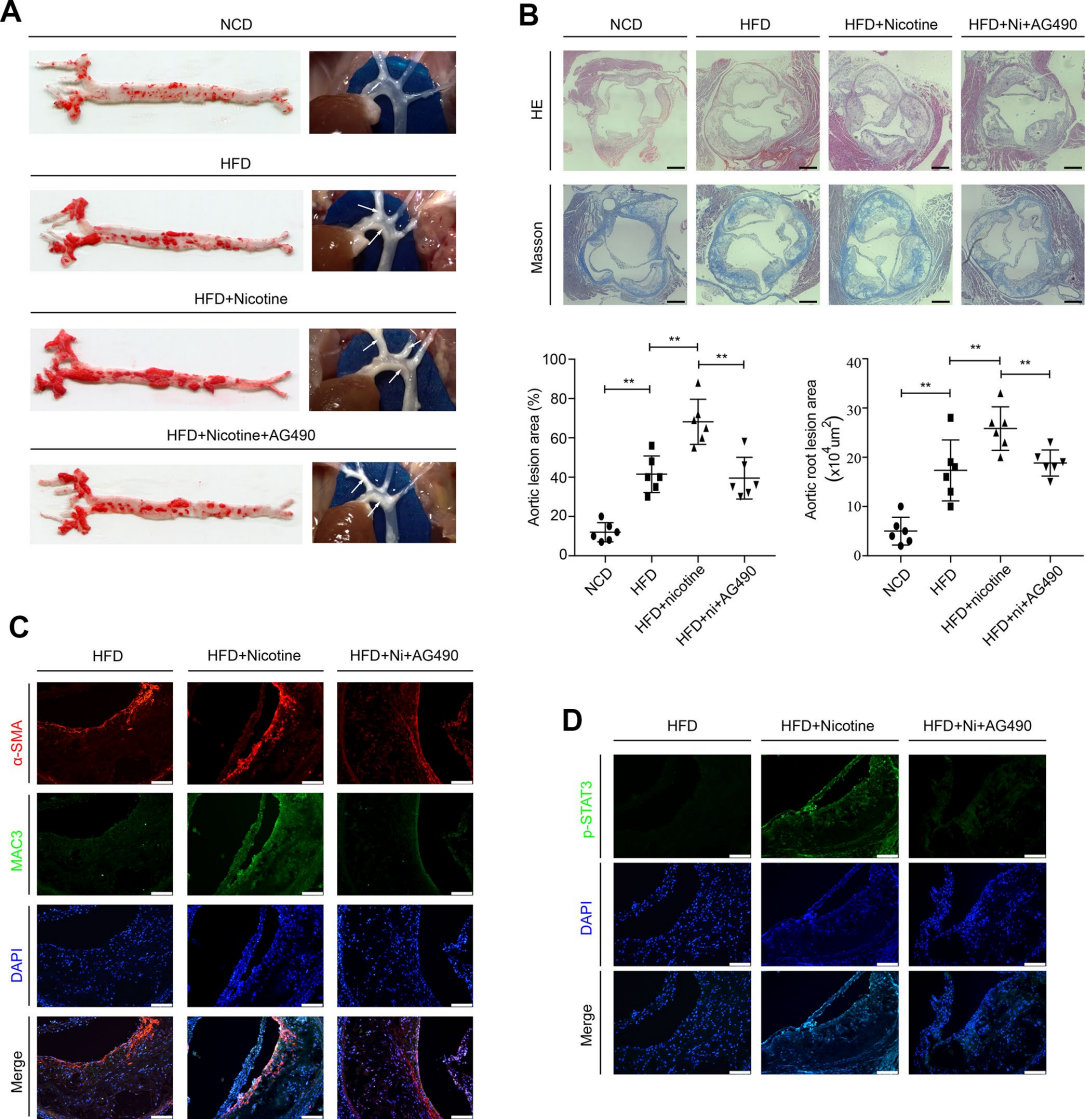
**Inactivation of STAT3 attenuates nicotine-induced plaque development in vivo.** (**A**) Lesions in en face aortas, as determined by oil red O staining and imaging. Arrow means atherosclerotic lesion. (**B**) Lesions in aortic root cross-sections, as determined by HE and Masson staining. Magnification, 100×; bars, 200 μm. (**C**) The components of atherosclerotic lesions were determined by a-SMA and Mac3 staining. Magnification, 200×; bars, 100 μm. (**D**) The expression of p-STAT3 in atherosclerotic lesions. Magnification, 200×; bars, 100 μm. Abbreviations: NCD, normal chow diet; HFD, high fat diet; Ni, nicotine. The data were presented as the mean ± SD, *p < 0.05, **p < 0.01 (n=6).

*ApoE^-/-^* mice were randomly assigned to the following 3 groups: the high fat diet (HFD) group, HFD + nicotine group, and HFD + nicotine + AG490 (STAT3 inhibitor) group. They were fed a high-fat diet (Research Diet, D12109C: 40 % fat, 1.25% cholesterol, and 0.5% cholic acid) for 12 weeks and given drinking water with or without dissolved nicotine. The STAT3 inhibitor AG490 was intraperitoneally injected into mice at a dosage of 100 μg for 12 weeks. *ApoE^-/-^* mice that were fed a normal chow diet (the NCD group) were also used to evaluate the successful establishment of the atherosclerotic model. After these feeding regimens, weight, systolic blood pressure (SBP) and serum lipid profiles were assessed. We observed no differences between the HFD group, HFD + nicotine group, and the HFD + nicotine + AG490 group (Supplementary Table 1). At the end of treatment, we evaluated lesion development in en face aortas by oil red O staining. We found that the atherosclerotic lesion areas are significantly increased in the HFD + nicotine group compared with the NCD group, HFD group, and HFD + nicotine + AG490 group (Figure 6A). Additionally, HE and Masson staining was used to measure the lesion areas in aortic root cross-sections (Figure 6B), and the results were consistent with those of oil red O staining. We also evaluated the lesion components by Mac3 and α-smooth muscle actin (α-SMA) staining, and observed that the HDF + nicotine group has a higher VSMC and macrophage content than that of the other groups (Figure 6C). Furthermore, we observed that STAT3 phosphorylation in atherosclerotic plaques stimulated by nicotine is abolished by AG490 treatment (Figure 6D). Collectively, these results provided evidence that the inactivation of STAT3 attenuates nicotine-induced plaque development.

### nAChRα1 knockdown attenuates nicotine-induced STAT3 phosphorylation in vivo

**Figure 7:**
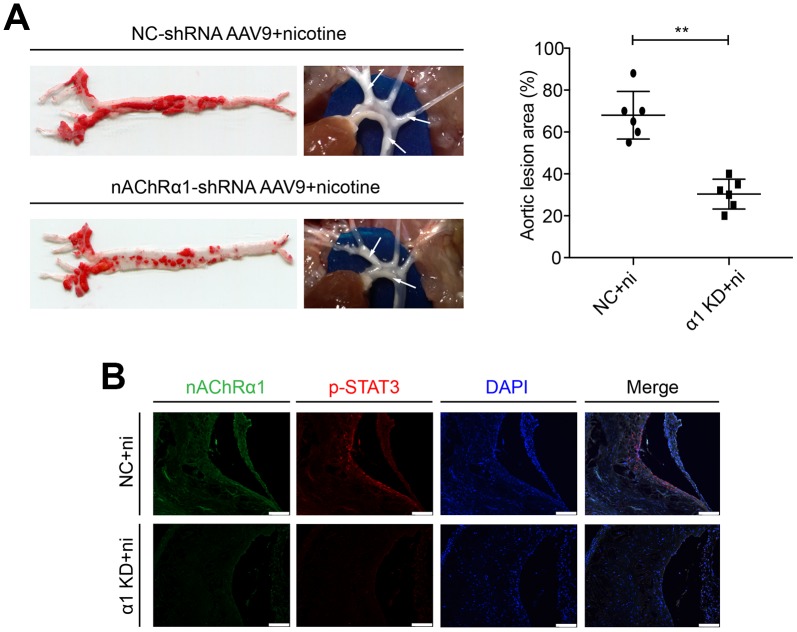
**nAChRα1 is involved in nicotine-induced atherosclerosis by mediating STAT3 phosphorylation in vivo.** (**A**) nAChRα1 knockdown attenuates nicotine-induced atherosclerotic progression in vivo. Arrow means atherosclerotic lesion. (**B**) nAChRα1 knockdown significantly decreases the nicotine-induced upregulation of p-STAT3. Magnification, 200×; bars, 100 μm. Abbreviations: α1 KD, nAChRα1 knockdown; NC, negative control; ni, nicotine. The data were presented as the mean ± SD, *p < 0.05, **p < 0.01 (n=6).

To determine whether nAChRα1 knockdown affects STAT3 activation in vivo, nAChRα1-shRNA-AAV9 and NC-shRNA-AAV9 were administered to the 8-week-old mice at a titer of 1 × 10^11^eg/ml in a 200 μl volume via the tail vein for one time. The mice were then fed a high-fat diet and drinking water with nicotine. After 12 weeks, we found that nAChRα1 knockdown significantly decreases nicotine-induced atherosclerotic progression (Figure 7A). Then, we detected the expression of nAChRα1 and p-STAT3 in atherosclerotic lesions in aortic root cross-sections by immunofluorescence staining. We observed that nAChRα1 knockdown remarkably reduces the expression of p-STAT3 (Figure 7B), which provided evidence for the interaction between nAChRα1 and STAT3 in vivo. The baseline data of *ApoE^-/-^* mice in these two groups were shown in Supplementary Table 2.

## DISCUSSION

Cigarette smoking is a leading risk factor for cardiovascular diseases. Nicotine, one of the major components of cigarette smoke, has been reported to promote atherosclerosis both in vivo and in vitro [7–9, 14, 20, 21]. Nicotine increases the expression of CD36, which is involved in oxidized LDL uptake and foam cell formation, in macrophages [22]. Such effects are very important in the pathophysiology of atherosclerosis. VSMCs and endothelial cells are also involved in atherogenesis, and nicotine enhances their ability to proliferate, migrate and form tubes [23, 24]. It is generally acknowledged that the pro-atherogenesis effect of nicotine is mediated mainly through nAChRs [7]. Nicotine acts as an agonist of nAChRs on VSMCs to trigger the ERK1/2 proliferative pathway, thus inducing cell proliferation and post-injury neointimal formation [25]. Wang and colleagues revealed that nicotine-induced autophagy accelerates atherosclerotic progression via the nAChRs/ROS/NF-κB pathway [26]. In her study, nicotine-induced autophagy promoted the transition of VSMCs from a contractile phenotype to a synthetic phenotype. In addition, nicotine functions by activating the α7 subunit in mast cells, and a deficiency in either mast cells or the α7 subunit diminishes nicotine-induced atherosclerosis [27]. Our previous studies also revealed that nicotine induces the upregulation of MMP2, MCP-1, VCAM-1 and RANTES in macrophages and VSMCs through nAChRα7 [19, 28], and the knockdown of the nAChRα1 gene attenuates nicotine-induced plaque progression in vivo [14]. Our in vivo study also revealed a similar phenomenon, validating the effects of nicotine and nAChRα1 on atherogenesis. Moreover, we silenced and overexpressed nAChRα1 in MOVAS and RAW264.7 cells, and found that STAT3, Akt and mTOR, but not ERK1/2, might be the downstream molecules of nAChRα1.

STAT3 is a transcription factor that mediates extracellular signals involved in various cellular responses, such as apoptosis and inflammation [15]. A previous study revealed the complicated connection between STAT3 and atherosclerosis [16]. This study demonstrated that the atherosclerotic lesion area in the aortic roots in STAT3 knockout mice is significantly reduced compared with that in control mice, suggesting the importance of STAT3 signaling in the progression of atherosclerosis. In addition, the activation of JAK2/STAT3 by IL-1β leads to the proliferation and migration of VSMCs and neovascularization [29], while the inhibition of STAT3 phosphorylation has the opposite effects [30]. Furthermore, STAT3 inactivation downregulates the expression of vascular endothelial growth factor (VEGF) in human umbilical vein endothelial cells (HUVECs), thus decreasing VEGF-induced proliferation and migration and delaying the process of atherosclerotic plaque formation [31]. It has been reported that myocardial infarction-related transcript (MIAT) promotes cell proliferation in an atherosclerosis model by regulating the miR-181b/STAT3 axis [32]. miR-19b inhibits the angiogenesis of endothelial cells by inhibiting STAT3, thus reducing the progression of unstable plaques in patients with atherosclerosis [33]. Consistent with these studies, our results showed that the inhibition of STAT3 phosphorylation interferes with nicotine-induced atherosclerosis in vivo. The data also indicated that STAT3 inactivation remarkably attenuates the nicotine-induced proliferation and migration of VSMCs and inflammation in macrophages. Now here, we observed stronger staining for phospho-STAT3 (Tyr-705) in atherosclerotic lesions in the control group compared with the nAChRα1 knockdown group. Remarkably, we uncovered for the first time that nAChRα1 directly binds STAT3 in MOVAS and RAW264.7 cells.

In response to cellular stimuli, the phosphorylation of STAT3 leads to dimerization, followed by its translocation to the nucleus, where it mediates several target genes involved in proliferation, migration and inflammation. The transcriptional regulatory activity of STAT3 is partly dependent on nuclear localization. Our study confirmed the nuclear translocation of STAT3 upon nicotine stimulation by confocal microscopy and western blotting. Observations in MOVAS and RAW264.7 cell lines showed that the suppression of STAT3 activation via AG490 decreases nicotine-enhanced p-Akt and p-mTOR expression. Thus, we hypothesized that Akt/mTOR might be downstream of STAT3. Using ChIP analysis, for the first time, we demonstrated that activated STAT3 binds to the Akt promoter region, further indicating that Akt is a direct target of STAT3.

In conclusion, our study unveiled that STAT3 inhibition attenuates nicotine-induced atherogenesis by decreasing the migration and proliferation of VSMCs and inflammation in macrophages via Akt/mTOR/MMP2 signaling pathway. In addition, we identified the binding between nAChRα1 and STAT3, and confirmed Akt as a direct target of STAT3. Collectively, these novel data revealed a dynamic transcriptional pathway for nicotine-induces atherosclerosis. Targeting nAChRα1/STAT3 therefore might be considered a new therapeutic strategy for alleviating atherosclerotic lesions induced by nicotine.

### Limitations

In this study, we have revealed the possibility of nicotine accelerating atherosclerosis via nAChRα1/STAT3/Akt/mTOR signaling pathway. Our findings potentially offer novel therapeutic approaches for nicotine-induced atherosclerosis, but whether smokers can really benefit from nAChRα1 or STAT3 blockade remains to be further studied. The road leading from STAT3 inhibitors to mature therapies for atherosclerosis remains long [34]. In addition, we are only beginning to understand the complex interplay between transcription process. We have focused on the transcriptional factor and related signaling pathway. However, whether or not nicotine alters gene transcription through histone modification of promoter regions, such as acetylation or methylation, remains to be addressed.

### Future directions

In perspective, more detailed mechanisms of transcriptional regulation by which nicotine induces atherosclerosis need be pointed out. Based on the literatures that nicotine promotes transcription process by increasing histone acetylation and decreasing histone methylation [35, 36], epigenetic modifications of the transcriptional binding site remains to be further studied. Furthermore, clinical trials are necessary to investigate whether nAChRα1 or STAT3 blockade can attenuate atherosclerotic progression induced by nicotine.

## MATERIALS AND METHODS

### Antibodies and reagents

Anti-STAT3 (CST, Boston, USA, 12640); anti-GAPDH (CST, 5174); anti-MMP2 (CST, 87809); anti-p-STAT3 (CST, 9145); anti-Akt (CST, 9272); anti-p-Akt (CST, 9271); anti-mTOR (CST, 2983); anti-p-mTOR (CST, 5536); anti-ERK1/2 (CST, 4696); anti-p-ERK1/2 (CST, 4376); anti-MMP2 (CST, 87809); anti-nAChRα1 (Santa Cruz Biotechnology, CA, USA, sc-65829); anti-CHRNA1 (Sangon Biotech, China, D160643); anti-MCP-1 (Abcam, Cambridge, UK, ab9002); anti-CD107b (BD, New York, USA, 553322); anti-α-SMA (CST, 19245) and GFP-tag (Proteintech, China, 66002-1-Ig) antibodies were used. An HRP-conjugated goat anti-rabbit secondary antibody (CST, 7074); an HRP-conjugated goat anti-mouse secondary antibody (CST, 7076); an Alexa Flour 488-conjugated goat anti-mouse secondary antibody (Beyotime Biotechnology, China, A0428); an Alex Flour 488-conjugated goat anti-rabbit secondary antibody (Beyotime, A0423); an Alex Flour 555-conjugated donkey anti-rabbit secondary antibody (Beyotime, A0453); a FITC-conjugated goat anti rat secondary antibody (Sangon, D110262); a mouse control IgG antibody (Sangon, D110503); a rabbit control IgG antibody (Sangon, D110502). Protein A/G magnetic beads (MCE, New Jersey, USA, HY-K0202); AG490 (MCE, HY-12000); rapamycin (MCE, HY-10219); an EdU kit (Ribobio, China, C-10310-1); Lipofectamine 3000 transfection reagent (Invitrogen, Carlsbad, USA, L3000015); high-glucose DMEM medium (Gibco, USA, 11965092); Opti-MEM (Gibco, 31985062); a SimpleChIP Plus Sonication Chromatin IP Kit (CST, 56383); DAPI (Beyotime, C1005); nAChRα1-shRNA-AAV9, NC-shRNA-AAV9 (Genomeditech, China) were also used.

### Animal model

Eight-week-old male *ApoE^-/-^* mice (C57BL/6 background) were purchased from GemPharmatech Company (Nanjing, China). All mice were fed a high fat diet (Research Diet, D12109C [37]) for 12 weeks to establish atherosclerotic model. The mice were randomly divided into five groups (n=10/group): the control group, nicotine group, nicotine + AG490 (a STAT3 inhibitor) group, NC-AAV9 + nicotine group, and α1-AAV9 + nicotine group. Nicotine was mixed in the drinking water at a concentration of 100 μg/ml [7, 27]. AG490 was intraperitoneally injected into mice at a dosage of 100 μg for 12 weeks. The viruses were administered to the 8-week-old mice at a titer of 1 × 10^11^-eg/ml in a 200-μl volume via the tail vein for one time. α1-AAV9 efficiently suppressed α1 receptor expression in vivo, and NC-AAV9 was used as a negative control for α1-AAV9. Meanwhile, the NCD group (mice that were fed a normal chow diet) was used to assess the reliability of the atherosclerotic model. All mice were maintained and handled under a protocol approved by the Animal Care and Use Committee of School of Medicine at Shanghai Jiao Tong University, and complied with the National Institute of Health Guide for the Care and Use of Laboratory Animals.

### Atherosclerotic lesion assay

Mice were euthanized and perfused with saline. The aortas and hearts were then isolated and fixed in 4% paraformaldehyde overnight at 4 °C. Paraffin-embedded cross-sections (4μm) of the organs were stained with hematoxylin and eosin (HE) and Masson’s trichrome to evaluate the lesion size and collagen content. The pinned aortas of six mice from each group were stained with oil red O to evaluate the overall severity of the atherosclerotic plaques.

### Immunohistology

Paraffin-embedded aortic root sections were stained with primary antibodies against nAChRα1, STAT3, CD107b (Mac3), and α-SMA and were then labeled using Alexa Flour 555/488 conjugated secondary antibodies.

### Cell culture and transfection

A mouse vascular smooth muscle cell line (MOVAS) and mouse macrophage cell line (RAW264.7) were purchased from American Type Culture Collection (ATCC, Rockville, MD, USA). MOVAS and RAW264.7 cells were cultured in high glucose DMEM (Thermo Scientific, Waltham, MA, USA) containing 100 units/ml of penicillin-streptomycin and 10% fetal bovine serum (FBS) and grown at 37 °C in a 5% CO_2_ incubator/humidified chamber. MOVAS and RAW264.7 cells were seeded into six-well plates. When they reached 70% confluence, the cells were transfected with nAChRα1 siRNA, negative control siRNA, nAChRα1 overexpression plasmid, or negative control plasmid using Lipofectamine 3000 transfection reagent in accordance with the manufacturer’s instructions. Forty-eight hours after transfection, the cells were treated with nicotine, and the levels of different kinds of protein were determined by western blotting. Gene silencing and overexpression were also monitored by western blotting.

### Western blot analysis

Western blotting was performed as described previously [38]. Briefly, cells were lysed in ice-cold lysis buffer supplemented with a protease and phosphatase inhibitor complex (NCM Biotech, China, P002). The protein was run on 7.5% or 10% SDS page gels and blotted onto PVDF membranes (Millipore, USA, IPVH00010) by wet transfer. After the blots were blocked in 5% skimmed milk for 1 h, they were first incubated with primary antibodies against STAT3 (1:1000), p-STAT3 (1:1000), Akt (1:1000), p-Akt (1:1000), mTOR (1:1000), p-mTOR (1:1000), ERK1/2 (1:1000), p-ERK1/2 (1:1000), MMP2 (1:1000), GAPDH (1:1000), and nAChRα1 (1:500) at 4 °C overnight, and then with the appropriate HRP-conjugated secondary antibodies (1:2000) for 1 h at room temperature. The immunoreactive bands were developed using the enhanced chemiluminescent method (NCM Biotech, China), and the signals were detected using the ChemiDoc XRS Plus luminescent image analyzer (Bio-Rad, USA). GAPDH was included as an internal loading control. The intensity of the western blot signals was quantified using ImageJ software (National Institutes of Health, Bethesda, MD, USA).

### Immunofluorescence staining

Cells cultured in 35-mm glass dishes (Cellvis, Mountain View, USA, D35-20-1.5P) were fixed with 4% paraformaldehyde for 20 min and permeated with 0.5% Triton X-100 for 30 min. The cells were then blocked with 2% BSA for 1 h and probed with anti-MCP-1 (1:100), anti-STAT3 (1:100), anti-p-STAT3 (1:100), and anti-nAChRα1 (1:50) antibodies at 4 °C overnight. After washing 3 times with PBST, they were incubated with Alex Flour 488/555- or FITC-conjugated secondary antibodies at room temperature for 2 h. Then, they were washed 3 times with PBST, and DAPI solution was added to stain the cell nucleus. The dishes were analyzed by laser confocal microscopy with excitation at 488 nm and 555 nm. ImageJ software was used for quantitative analysis, and total antibody staining was normalized to DAPI.

### Cell proliferation assay

An EdU kit was used for the cell proliferation assays. A total of 10000 cells were seeded into 24-well plates and incubated overnight. After treatment with nicotine (0 ng/ml, 10 ng/ml, 100 ng/ml or 1000 ng/ml) for 24h, the cell medium was replaced with medium containing EdU (final concentration of 50 μM) for 2 h at 37°C. Subsequently, the cells were fixed with 4% formaldehyde for 20 min and permeated with 0.5% Triton X-100 for 15 min. The cells were then treated with an Apollo reaction cocktail for 30 min and Hoechst 33342 for 15 min at room temperature. The images were captured with a fluorescence microscope (Leica) and analyzed using ImageJ software. The EdU incorporation rate was expressed as the ratio of EdU-positive cells to the total number of cells in each field.

### Transwell migration assay

Twenty-four-well transwell chambers with 8-μm microporous membranes (Corning, Beijing, China) were used to perform the migration assay. Cell suspensions (0.1 ml of FBS-free medium, 2×10^5^ cells/ml) were added to the upper chamber and incubated for 24 h at 37°C. The lower chamber contained DMEM supplemented with 10% FBS. Nicotine and other inhibitors were added to both chambers. After incubation, the cells were fixed with methanol for 20 min and stained with 0.5% crystal violet solution for 30 min. Non-migrating cells were removed from the upper surface of the insert using cotton swab and migrating cells were quantified by light microscopy. The number of migrated cells was counted in three random fields per chamber and normalized to the corresponding control.

### CoIP

MOVAS and RAW264.7 cells were washed in PBS. The cells were lysed in ice-cold PBST (0.5% Triton X-100 in PBS) supplemented with a protease and phosphatase inhibitor complex. Whole cell lysates (400 μl) were incubated with an anti-STAT3 antibody (2 μl), an anti-nAChRα1 antibody (30 μl), and normal mouse IgG (5 μl) for 2 h at room temperature. A 30-μl protein A/G magnetic bead slurry was transferred to a 1.5-ml tube, and the beads were washed 3 times with PBST. Then, the beads were added to whole-cell lysates and mixed for 2 h at room temperature. After washing 3 times with PBST, the beads were boiled, eluted in SDS loading buffer and analyzed by western blotting. In addition, α1-GFP-plasmids or STAT3-GFP-plasmids were transfected into 293T cells to confirm binding. An anti-GFP-tag antibody (5 μl) was used, and the other steps were the same.

### ChIP-PCR

Cells were treated with or without nicotine (100 ng/ml) for 6 h and cross-linked in 1% formaldehyde for 15 min [39, 40]. After cell lysis, the chromatin was fragmented to 200-1000 bp by sonication, and enriched with antibodies against p-STAT3 or isotype IgG at 4°C overnight. Protein G Magnetic Beads were added to each IP reaction and incubated at 4°C for 2 h. Then, the chromatin was eluted from the beads, and the cross-links were reversed by NaCl and proteinase K before DNA purification. The purified DNA was subjected to polymerase chain reaction (PCR). The amount of immunoprecipitated DNA was normalized to the input DNA. The binding sites were predicted by JASPAR (http://jaspar.genereg.net) with a relative profile score threshold of 80% and the following primer sequences of Akt were used: forward: 5′-AGGTGTGTAGCCACTTGATGACTAT-3′; reverse: 5′-GGTTCCTTAGCTGGGGTGAAT-3’.

### ELISA and lipid assay

The level of inflammatory cytokines in the cell culture supernatant were measured with an ELISA Kit (Multisciences, China) according to the manufacturer’s instructions. Serum total cholesterol, triglyceride, low-density lipoprotein cholesterol (LDL-C) and high-density lipoprotein cholesterol (HDL-C) levels were tested by Olympus AU540.

### Statistical analysis

The values are presented as the mean ± standard deviation (SD). All data were from at least three independent experiments, each of which was performed in triplicate. Statistical analyses were performed using SPSS 21.0 software. The data were analyzed by Student’s *t*-test or by ANOVA, followed by the Bonferroni post-hoc test. *p*<0.05 was considered statistically significant.

## Supplementary Material

Supplementary Figures

Supplementary Tables
