# Supplementary material for: The interaction between STAT3 and nAChRα1 interferes with nicotine-induced atherosclerosis via Akt/mTOR signaling cascade
Source: Aging (Albany NY). 2019 Oct 14;11(19):8120–38. doi: 10.18632/aging.102296 (PMC6814582; doi:10.18632/aging.102296)
Supplement: Supplementary Figures [file aging-11-102296-s002.pdf]

SUPPLEMENTARY FIGURES

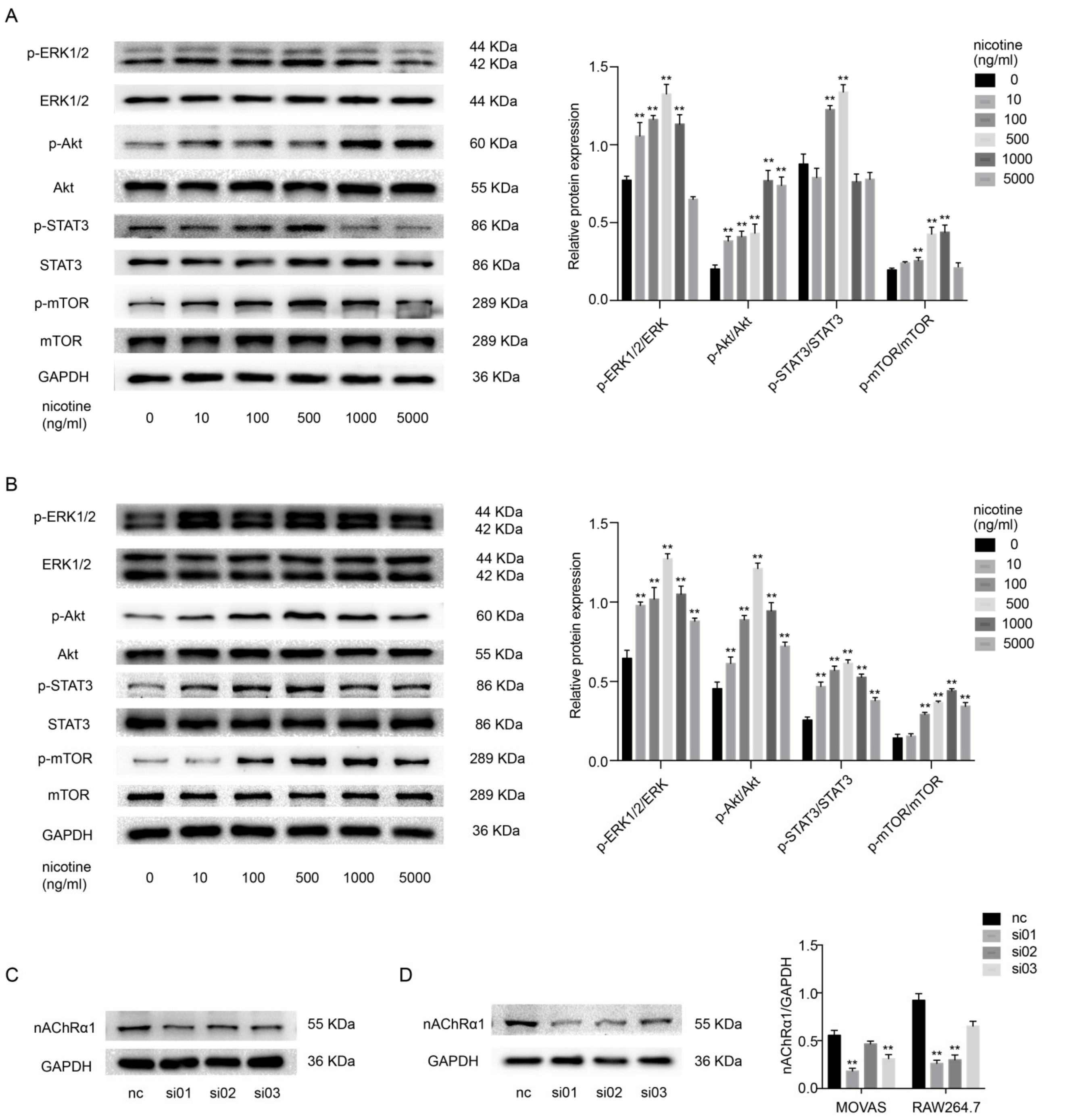

Supplementary Figure 1. The determination of the optimal nicotine concentration and siRNA targeting the nAChRα1 gene. (**A**) Nicotine stimulated the phosphorylation of STAT3, ERK1/2, Akt and mTOR in MOVAS cells. Two cell lines were treated with nicotine at the concentration of 0, 10, 100, 500, 1000, 5000ng/ml for 6 hours. The expression and activation of STAT3, ERK1/2, Akt and mTOR were detected by western blot. (**B**) Nicotine enhanced the activation of STAT3, ERK1/2, Akt and mTOR in RAW264.7 cells. (**C**) The effect of the siRNA targeting the nAChRα1 gene in MOVAS cells. (**D**) The effect of siRNA targeting nAChRα1 gene in RAW264.7 cells. Data were presented as mean ± SD. *$p < 0.05$, **$p < 0.01$ *vs*. the control group. Each experiment was performed three times.

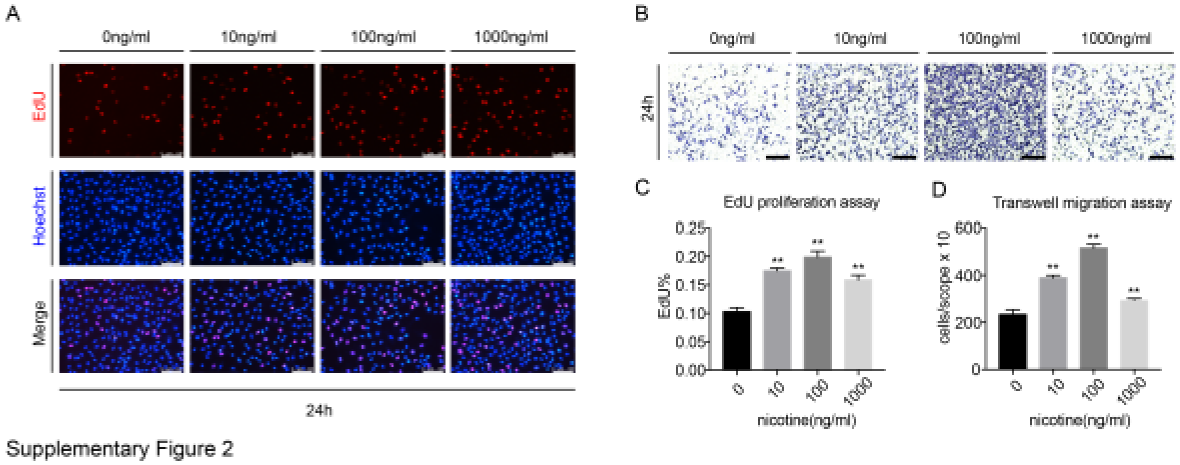

Supplementary Figure 2. Nicotine promotes the migration and proliferation of MOVAS cells in a concentration-dependent manner. (**A**) The proliferation of MOVAS cells under different concentrations of nicotine exposure detected by EdU assay. Magnification, 200×; bars, 100 μm. (**B**) The migration of MOVAS cells under different concentrations of nicotine exposure detected by Transwell assay. Magnification, 100×; bars, 250 μm. (**C**) The statistical analysis of Edu assay. (**D**) The statistical analysis of Transwell assay. Data were presented as mean ± SD. *$p < 0.05$, **$p < 0.01$ *vs*. the control group. Each experiment was performed three times.
