# Supplementary material for: The interaction between STAT3 and nAChRα1 interferes with nicotine-induced atherosclerosis via Akt/mTOR signaling cascade
Source: Aging (Albany NY). 2019 Oct 14;11(19):8120–38. doi: 10.18632/aging.102296 (PMC6814582; doi:10.18632/aging.102296)
Supplement: Supplementary Tables [file aging-11-102296-s001.pdf]

SUPPLEMENTARY TABLES

Supplementary Table 1. The baseline characteristics of *ApoE*$^{-/-}$ mice.

	NCD	HFD	HFD+Nicotine	HFD+Nicotine+AG490
Weight gain, g	13.32±0.373	5.78±0.872*	6.08±0.664*	4.77±0.874*&
SBP, mmHg	99.73±4.945	104.14±7.126	108.28±4.030*	106±5.195*
Triglycerides, mmol/L	0.966±0.304	0.789±0.201*	0.791±0.221	0.801±0.192
Total cholesterol, mmol/L	18.128±3.018	34.376±3.174*	36.124±4.769*	35.927±2.981*
HDL-C, mmol/L	1.709±0.183	2.833±0.327*	2.847±0.402*	2.862±0.318*
LDL-C, mmol/L	9.263±0.635	8.128±0.609	8.603±0.647	9.017±0.597
Fast blood glucose, mmol/L	5.64±0.905	7.26±1.025*	7.37±0.843*	7.43±0.709*
Blood uric acid, µmol/L	95.847±26.328	108.932±36.477*	121.004±52.937*#	112.382±35.250*

NCD, Normal chow diet, HFD, High-fat diet. SBP, systolic blood pressure; HDL-C, high-density lipoprotein cholesterol; LDL-C, low-density lipoprotein cholesterol. Data were presented as mean ± SD, *p <0.01 vs. the NCD group, #p<0.05 vs. the HDF group, &p<0.05 vs. the HFD+Nicotine group. (n=10).

Supplementary Table 2. The baseline characteristics of *ApoE*$^{-/-}$ mice.

	NC-AAV9+Nicotine	α1-AAV9+Nicotine
Weight gain, g	5.07±0.812	5.42±0.657
SBP, mmHg	100.30±5.623	105.25±3.980
Triglycerides, mmol/L	0.741±0.182	0.825±0.235
Total cholesterol, mmol/L	37.248±3.081	35.025±5.137
HDL-C, mmol/L	2.824±0.138	2.853±0.375
LDL-C, mmol/L	8.355±0.679	9.040±0.621
Fast blood glucose, mmol/L	7.13±0.509	7.36±0.497
Blood uric acid, µmol/L	110.382±42.352	115.420±28.774

SBP, systolic blood pressure, HDL-C, high-density lipoprotein cholesterol, LDL-C, low-density lipoprotein cholesterol. were presented as mean ± SD, *p<0.05 vs. the NC-AAV9+Nicotine group. (n=10).
